# Physiological and transcriptomic analyses reveal the mechanisms underlying the salt tolerance of *Zoysia japonica* Steud

**DOI:** 10.1186/s12870-020-02330-6

**Published:** 2020-03-14

**Authors:** Jingjing Wang, Cong An, Hailin Guo, Xiangyang Yang, Jingbo Chen, Junqin Zong, Jianjian Li, Jianxiu Liu

**Affiliations:** Institute of Botany, Jiangsu Province and Chinese Academy of Sciences, Nanjing, 210014 China

**Keywords:** DUF, Hormone signal, Salt tolerance, Transcription factor, Transcriptome, *Zoysia* grass

## Abstract

**Background:**

Areas with saline soils are sparsely populated and have fragile ecosystems, which severely restricts the sustainable development of local economies. *Zoysia* grasses are recognized as excellent warm-season turfgrasses worldwide, with high salt tolerance and superior growth in saline-alkali soils. However, the mechanism underlying the salt tolerance of *Zoysia* species remains unknown.

**Results:**

The phenotypic and physiological responses of two contrasting materials, *Zoysia japonica* Steud. Z004 (salt sensitive) and Z011 (salt tolerant) in response to salt stress were studied. The results show that Z011 was more salt tolerant than was Z004, with the former presenting greater K^+^/Na^+^ ratios in both its leaves and roots. To study the molecular mechanisms underlying salt tolerance further, we compared the transcriptomes of the two materials at different time points (0 h, 1 h, 24 h, and 72 h) and from different tissues (leaves and roots) under salt treatment. The 24-h time point and the roots might make significant contributions to the salt tolerance. Moreover, GO and KEGG analyses of different comparisons revealed that the key DEGs participating in the salt-stress response belonged to the hormone pathway, various TF families and the *DUF* family.

**Conclusions:**

*Zoysia* salt treatment transcriptome shows the 24-h and roots may make significant contributions to the salt tolerance. The auxin signal transduction family, ABA signal transduction family, *WRKY* TF family and *bHLH* TF family may be the most important families in *Zoysia* salt-stress regulation.

## Background

Soil salinization is a worldwide problem. Areas with saline soils are sparsely populated and have fragile ecosystems, which severely restricts the sustainable development of local economies. As an important part of landscaping, turf plays an important role in protecting, improving and beautifying urban environments. Therefore, it is particularly important to choose high-quality salt-tolerant turfgrass suitable for landscaping in areas with saline soils. *Zoysia* Willd. is a genus of perennial plants belonging to the family *Poaceae*, subfamily *Chloridoideae*, tribe *Zoysieae* [51]. *Zoysia* grasses are recognized as excellent warm-season turfgrasses worldwide; they are with salt tolerant, hardy, and drought tolerant and are widely used in athletic fields, home lawns and parks [10]. Compared with other *Poaceae* family members, *Zoysia* grasses have received less attention in the research community. However, as an alternative grass species for landscaping in saline-alkali soil, *Zoysia* has superior growth qualities [26]. In particular, among the three most important commercial species, *Zoysia japonica* Steud. is distinctly tolerant to abiotic stress [51]. Therefore, studying the salt tolerance of *Zoysia* plants is highly important.

Previous studies on salt tolerance of *Zoysia* mainly focused on the evaluation of salt tolerance and the physiological mechanisms governing salt tolerance. Salt tolerance evaluations have shown that the salt tolerance of *Zoysia* plants has rich genetic variation [25, 35, 40, 57]. This variation makes for convenient selection of materials with contrasting salt tolerances for studying the salt tolerance mechanism of *Zoysia*. *Zoysia* plants secrete salt; all *Zoysia* plant leaves have salt glands that regulate ion balance by selectively secreting salt ions. The salt tolerance of *Zoysia* plants is positively correlated with the rate of Na^+^ secretion from salt glands in leaves and the density of salt glands per unit leaf area [21, 22, 33]. Moreover, previous studies have shown that the salt tolerance of *Zoysia* is negatively correlated with the content of Na^+^ and positively correlated with the content of K^+^ in the leaf fluid. Salt-tolerant materials have a strong ability to maintain the K^+^/Na^+^ ratio in their leaves and roots. The Na^+^ content in leaves has been successfully used to evaluate the salt tolerance of *Zoysia* [25, 33, 34].

The salt tolerance of *Zoysia* is a very important trait, but to date, its molecular regulatory mechanism remains unknown. The Na^+^/H^+^ antiporter gene *ZjNHX1*, which belongs to the plant NHX-gene family, was cloned from *Z. japonica*, and studies have shown that *ZjNHX1* plays an important role in ion homeostasis and salt tolerance [9]. In addition, the glycine-rich RNA-binding protein-coding gene *ZjGRP* was isolated from *Z. japonica* and was strongly induced by NaCl treatment. *ZjGRP*-overexpressing *Arabidopsis thaliana* plants present low germination rates, slow seedling growth and poor salt tolerance [50]. *ZjZFN1* is a C_2_H_2_-type zinc finger protein-coding gene that is expressed more in leaf tissues than in root and stem tissues, and its expression is induced by salt, cold and abscisic acid (ABA) treatments. Overexpressing *ZjZFN1* in *A. thaliana* can improve seed germination and increase salt tolerance by improving the transcriptional activities of several salt-tolerance-related genes under salt stress [49].

Studies on the salt tolerance genes of *Zoysia* are scarce. However, using a full-length cDNA expression library in yeast, Chen et al. [5] systematically excavated the salt tolerance genes in *Zoysia matrella* and identified 16 candidate salt tolerance genes involved in ion regulation, osmotic adjustment, protein folding and modification, mitochondrial membrane translocase and RNA metabolism. Xie et al. [58] presented the first comprehensive transcriptome data of *Z. japonica* Steud. roots, and a total of 32,849 unigenes and 4842 simple sequence repeats (SSRs) were identified. Their results showed that transcription factors (TFs) including members of the *AP2/EREBP* family, *bZIP* family, *NAC* family, *WRKY* family, *MYB* family and *bHLH* family play significant roles in the early response to salt stress [58].

Studies of the salt tolerance of zoysiagrass so far have focused on evaluating the salt tolerance among different cultivars, the physiological mechanisms of salt tolerance and the development of molecular markers [11, 59]. However, the molecular mechanism of salt tolerance in zoysiagrass remains unclear. In this study, we investigated the phenotypic and physiological responses of two materials with contrasting salt tolerances, *Z. japonica* Z004 (salt sensitive) and Z011 (salt tolerant), in response to salt stress. On the basis of the existing *Zoysia* reference genome [48], the HiSeq™ 2000 platform was used to perform RNA sequencing (RNA-seq) of the zoysiagrass leaves and roots. We then compared the transcriptomes at different time points (0 h, 1 h, 24 h, and 72 h) and of different tissues (leaves and roots) under salt treatments to identify the significant time points and tissues. According to the Gene Ontology (GO) and Kyoto Encyclopedia of Genes and Genomes (KEGG) analyses of differentially expressed genes (DEGs) in different comparisons, the key DEGs participating in the salt-stress response were selected, and these DEGs belonged to the hormone pathway, TF families and the *DUF* family. Thus, our research provides fundamental information for use in future salt-stress studies of *Zoysia* and improves the understanding of molecular mechanisms in salt-tolerant plants.

## Results

### Phenotypic and physiological responses of *Z. japonica* Steud. To salt stress

**Fig. 1:**
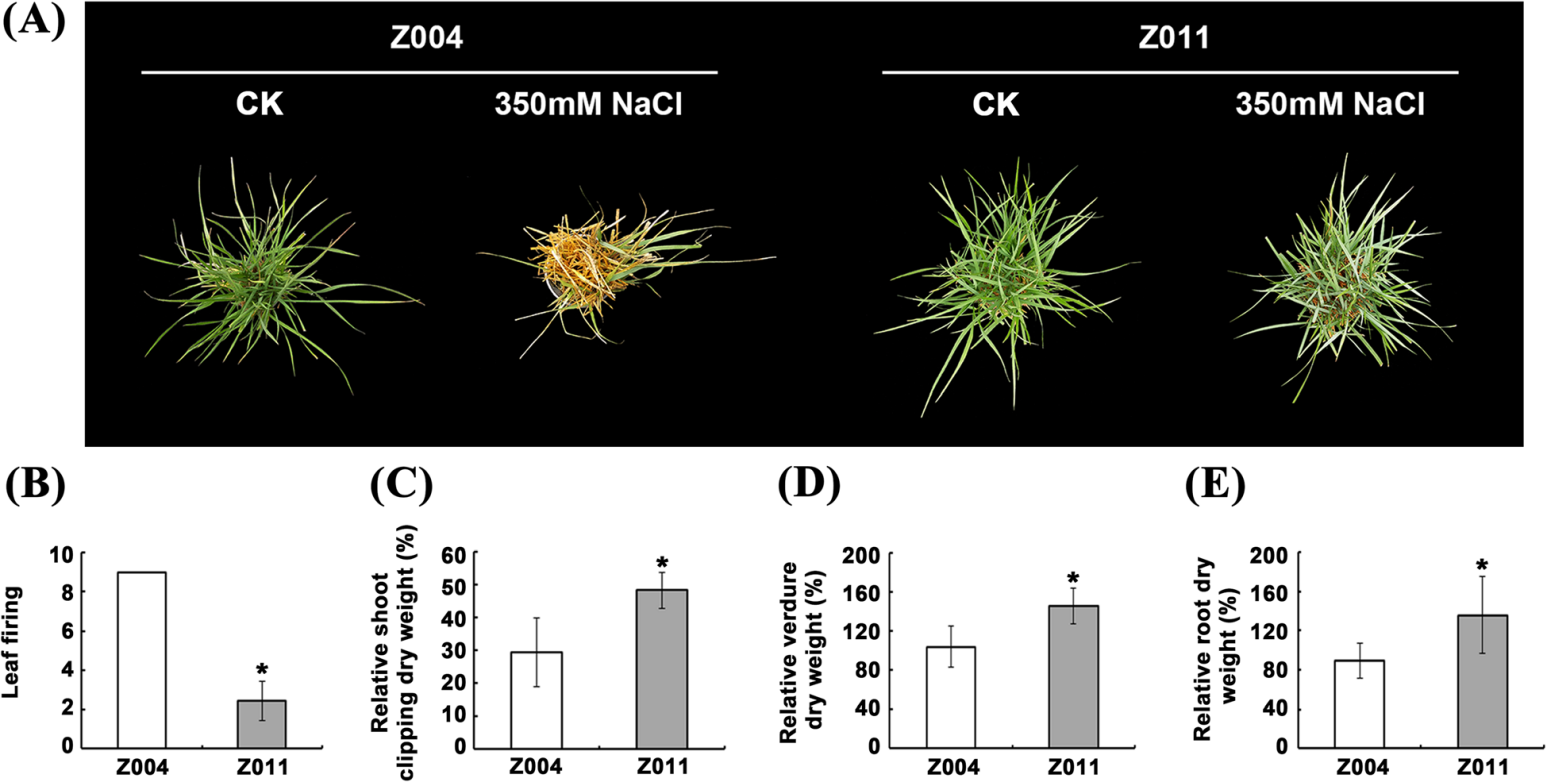
Phenotypic response of *Z. japonica* Steud. to salt stress. **a** Two materials with contrasting salt tolerances, Z004 (salt sensitive) and Z011 (salt tolerant), were exposed to 350 mM NaCl for 40 days. **b** The leaf firing of the Z004 and Z011 grasses after NaCl treatment for 40 days. **c** The relative shoot clipping dry weights of Z004 and Z011 after NaCl treatment for 40 days. **d** The relative verdure dry weights of Z004 and Z011 after NaCl treatment for 40 days. **e** The relative root dry weights of Z004 and Z011 after NaCl treatment for 40 days. The values are presented as the means ± SEs. The asterisks above the bars indicate significant differences between the respective values (*p* < 0.05)

Japanese lawngrass (*Z. japonica* Steud.) is a popular and important warm-season turfgrass, and different accessions have different degrees of salt tolerance. In this study, two accessions with contrasting salt tolerances, Z004 (salt sensitive) and Z011 (salt tolerant), were chosen to analyse the salt tolerance mechanism of *Z. japonica*. The salt treatment results showed that Z011 had strong salt tolerance and displayed good growth, while Z004 was sensitive to salt and withered and yellowed after treatment with 350 mM NaCl for 40 days (Fig. 1a). Moreover, the leaf firing of Z004 was significantly greater than that of Z011 (Fig. 1b), and the biomass statistics showed that the relative shoot clipping dry weight, verdure dry weight and root dry weight of Z011 were markedly greater than those of Z004 (Fig. 1c-e).

**Fig. 2:**
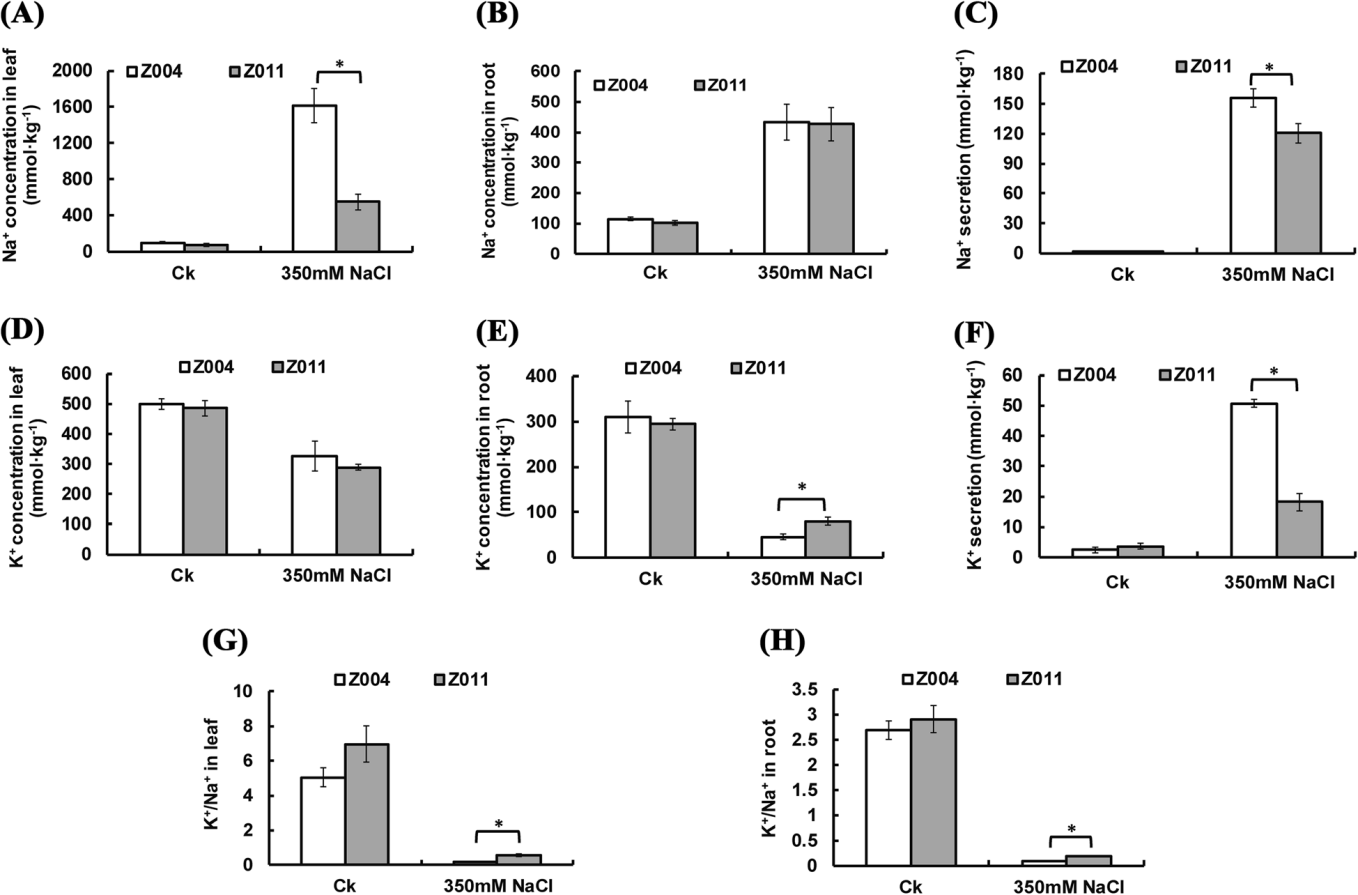
Physiological response of *Z. japonica* Steud. to salt stress. **a** The Na^+^ concentration in Z004 and Z011 leaves after Ck and NaCl treatment. **b** The Na^+^ concentration in Z004 and Z011 roots after Ck and NaCl treatment. **c** Na^+^ secretion by Z004 and Z011 after Ck and NaCl treatment. **d** The K^+^ concentration in Z004 and Z011 leaves after Ck and NaCl treatment. **e** The K^+^ concentration in Z004 and Z011 roots after Ck and NaCl treatment. **f** K^+^ secretion by Z004 and Z011 after Ck and NaCl treatment. **g** The K^+^/Na^+^ ratio in Z004 and Z011 leaves after Ck and NaCl treatment. **h** The K^+^/Na^+^ ratio in Z004 and Z011 roots after Ck and NaCl treatment. The values are presented as the means ± SEs. The asterisks above the bars indicate significant differences between the respective values (*p* < 0.05)

To study the differences in the mechanism of salt tolerance between Z004 and Z011, the Na^+^ and K^+^ concentrations were measured in the leaves, roots and secretions. In the control (CK) group, the Na^+^ concentrations and K^+^ concentrations in the leaves, roots and secretions were not significantly different between Z004 and Z011 (Fig. 2a-f). After treatment with 350 mM NaCl, the Na^+^ concentrations in the leaves, roots and secretions of Z004 and Z011 were greater than those in the CK (Fig. 2a-c). In the roots of Z004 and Z011 after NaCl treatment, the Na^+^ concentrations were not different (Fig. 2b). However, in the leaves, the Na^+^ concentrations and secretions were significantly lower in Z011 than in Z004 (Fig. 2a, c).

After treatment with 350 mM NaCl, the K^+^ concentrations in the leaves of Z004 and Z011 were lower than those in the leaves of the CK, but there were no differences in the K^+^ concentrations between Z004 and Z011 (Fig. 2d). In addition, the K^+^ concentrations in the roots of Z004 and Z011 were lower than those in the roots of the CK, and the K^+^ concentration in Z011 was significantly greater than that in Z004 (Fig. 2e). However, the K^+^ secretion in Z004 and Z011 after treatment with NaCl was greater than that in the CK, and the K^+^ secretion of Z004 was significantly greater than that of Z011 (Fig. 2f). Comparing with Z004, Z011 maintained a greater K^+^/Na^+^ ratio in both the leaves and roots (Fig. 2g, h).

### Transcriptome sequencing of the Z004 and Z011 accessions

**Fig. 3:**
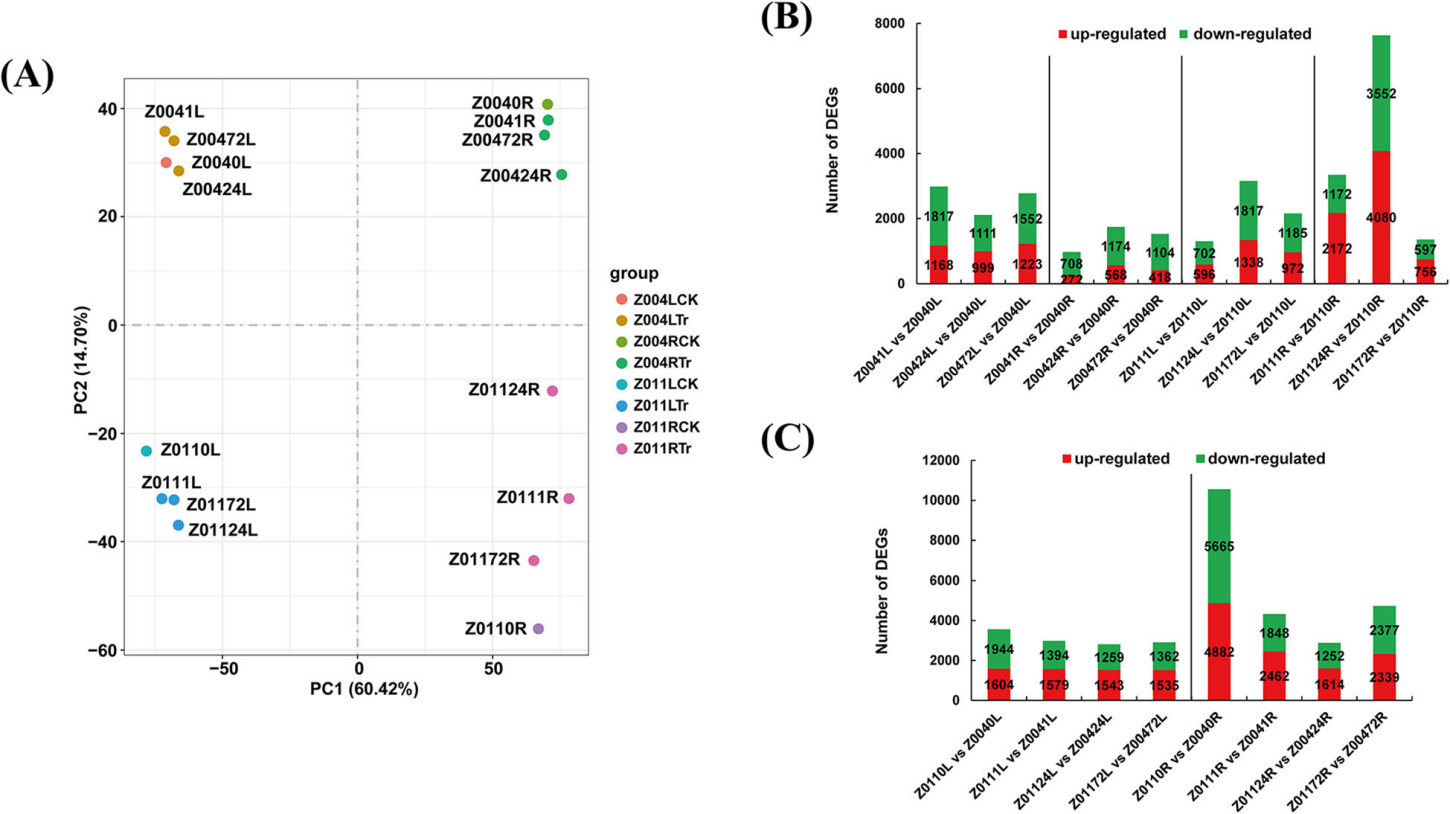
Expression profiles of *Z. japonica* salt tolerance-related transcripts. **a** PCA analysis of 16 transcriptome samples including four time points (0 h, 1 h, 24 h, and 72 h) and two tissues (leaves and roots) of Z004 and Z011 under salt treatment. The 0-h time point represents the CK group, and the 1-h, 24-h and 72-h time points constitute the Tr group. **b** The number of up- and downregulated DEGs in Z0041L vs Z0040L, Z00424L vs Z0040L, Z00472L vs Z0040L, Z0041R vs Z0040R, Z00424R vs Z0040R, Z00472R vs Z0040R, Z0111L vs Z0110L, Z01124L vs Z0110L, Z01172L vs Z0110L, Z0111R vs Z0110R, Z01124R vs Z0110R, Z01172R vs Z0110R. **c** The number of up- and downregulated DEGs in Z0110L vs Z0040L, Z0111L vs Z0041L, Z01124L vs Z00424L, Z01172L vs Z00472L, Z0110R vs Z0040R, Z0111R vs Z0041R, Z01124R vs Z00424R and Z01172R vs Z00472R

Leaf and root samples for RNA-seq were collected at 0 h, 1 h, 24 h and 72 h after treating Z004 and Z011 with 350 mM NaCl. In total, 16 samples were sequenced on the HiSeq™ 2000 sequencing platform. We obtained an average of 28.8 million raw reads from the 16 libraries, and 97.18% of the sequences were confirmed as clean reads (Online Resource 1). First of all, the total reads of our RNA-seq were mapped to the rice and sorghum genomes as references via Hisat2 (v2.0.5) software [17]. The results showed that the total reads to the rice reference genome (ftp://ftp.ncbi.nlm.nih.gov/genomes/all/GCF/001/433/935/GCF_001433935.1_IRGSP-1.0) was 0.12–0.39% (Online Resource 7), and the total reads to the sorghum reference genome (ftp://ftp.ncbi.nlm.nih.gov/genomes/all/GCF/000/003/195/GCF_000003195.3_Sorghum_bicolor_NCBIv3) was 0.26–0.85% (Online Resource 8). Afterward, the clean reads were mapped to the whole *Z. japonica* genome, and 62.7–91.39% of the total reads and 61.8–89.84% of the unique reads were mapped to the reference genome. Therefore, it is appropriate to select the zoysia genome as a reference. In addition, multiple-mapped reads constituted 0.85–1.67% of the total reads, and splice-mapped reads constituted 17.58–32.42% of the total reads. Furthermore, 73.44–77.17% of the total reads were mapped to exons in the reference genome, 13.38–16.10% of the reads were mapped to introns, and 8.58–12.06% of the reads were mapped to intergenic regions (Online Resource 2). In total, 59,271 unigenes and 29,675 novel genes were revealed by the RNA-seq assays. There were more genes (62,172, 69.90%) with lengths exceeding 1000 bp than those (21,909, 24.63%) with lengths exceeding 300 bp and fewer than 1000 bp. For the convenience of comparison, we defined 0 h as the CK group and 1 h, 24 h and 72 h as the treatment (Tr) group. Principal component analysis (PCA) revealed that the leaf and root samples of Z004 and Z011 were separated into four different areas (Fig. 3a). Among them, the Z011 root samples had the highest dispersion degree (Fig. 3a). Moreover, the 24 h samples of the roots of Z004 and Z011 were separated from the other samples, indicating that 24 h could be a crucial time (Fig. 3a).

### Identification of DEGs in Z004 and Z011 in response to salt stress

To characterize the differences between Z004 and Z011 in response to salt stress, we explored the unigenes whose expression level significantly changed after NaCl treatment. The transcript abundance of each gene was calculated by their fragments per kilobase per million fragments (FPKM) values. edgeR software [41] was used to analyse the significant differences in expression with padj < 0.05 and fold change > 2, and DEGs were identified as having a |log2(fold change)| > 1 and padj < 0.05. Under salt stress conditions, a total of 4701 genes, 2591 genes, 4400 genes and 8846 genes were differentially expressed in the leaves of Z004 (Z0041L vs Z0040L, Z00424L vs Z0040L, Z00472L vs Z0040L), Z004 roots (Z0041R vs Z0040R, Z00424R vs Z0040R, Z00472R vs Z0040R), Z011 leaves (Z0111L vs Z0110L, Z01124L vs Z0110L, Z01172L vs Z0110L) and Z011 roots (Z0111R vs Z0110R, Z01124R vs Z0110R, Z01172R vs Z0110R), respectively. In addition, a total of 12,220 genes were differentially expressed in the leaf comparisons of Z011 and Z004 (Z0110L vs Z0040L, Z0111L vs Z0041L, Z01124L vs Z00424L, Z01172L vs Z00472L), and 22,439 genes were differentially expressed in the root comparisons of Z011 and Z004 (Z0110R vs Z0040R, Z0111R vs Z0041R, Z01124R vs Z00424R, Z01172R vs Z00472R) (Fig. 3c). Interestingly, the number of DEGs of Z011 roots was obviously more than that in the Z004 roots, and the number of DEGs of roots was obviously more than leaves in comparisons of Z011 and Z004 (Fig. 3b,c). These results indicated that the roots might make a significant contribution to the difference in salt tolerance between Z004 and Z011. Moreover, comparisons of the Z004 leaves, Z004 roots and Z011 leaves revealed more downregulated genes than upregulated ones (Fig. 3b). However, in the comparisons of Z011 roots, there were more upregulated genes than downregulated ones (Fig. 3b). In addition, the number of DEGs in the 24-h sample (Z01124R vs Z0110R) was 2.28 and 5.64 times that in the 1-h (Z0111R vs Z0110R) and 72-h (Z01172R vs Z0110R) samples. Venn diagrams were constructed that also show that, in the comparisons of the Z004 roots, Z011 leaves and Z011 roots, the number of specific DEGs was greater at 24 h than at 1 h and 72 h (Supplementary Figure S1). These results indicated that 24 h might be a relatively important time point for the salt-stress response.

### GO analysis of DEGs in the Z004 and Z011 roots

For the functional characterization of DEGs, we assigned GO terms and selected significant GO classifications of DEGs in each comparison (padj < 0.05). Fourteen GO classes of ‘molecular function’ in the Z0041R vs Z0040R comparison were identified. In the comparison between Z00424R and Z0040R, 4 GO classes, 1 GO class and 20 GO classes fell into the categories ‘biological process’, ‘cellular component’ and ‘molecular function’ respectively. Meanwhile, in the Z00472R vs Z0040R comparison, the category ‘biological process’ had 2 GO classes, ‘cellular component’ had 1 GO class and ‘molecular function’ had 23 GO classes. In Z004R, 11 GO classes most relevant to the salt-stress response were concentrated in the Z00424R vs Z0040R comparison and Z00472R vs Z0040R comparison: ‘response to oxidative stress’, ‘response to stress’, ‘extracellular region’, ‘oxidoreductase activity, acting on paired donors, with incorporation or reduction of molecular oxygen’, ‘oxidoreductase activity, acting on peroxide as acceptor’, ‘antioxidant activity’, ‘peroxidase activity’, ‘iron ion binding’, ‘ubiquitin-protein transferase activity’, ‘ubiquitin-like protein transferase activity’ and ‘sequence-specific DNA binding’ (Online Resource 3). These results showed that Z004 responded slowly to salt stress. A significant stress response did not occur after 1 h of NaCl treatment but did occur after 24 h and 72 h.

Six GO classes of ‘molecular function’ in the Z0041R vs Z0040R comparison were identified. In the comparison between Z00424R and Z0040R, 4 GO classes, 1 GO class and 20 GO classes fell into the categories ‘biological process’, ‘cellular component’ and ‘molecular function’ respectively. Meanwhile, in the Z00472R vs Z0040R comparison, the category ‘biological process’ had 2 GO classes, ‘cellular component’ had 1 GO class and ‘molecular function’ had 23 GO classes.

In the comparison between Z0111R and Z0110R, 6 GO classes, 16 GO classes and 10 GO classes fell into the category ‘biological process’, ‘cellular component’ and ‘molecular function’ respectively. In the comparison between Z01124R vs Z0110R, 10 and 5 GO classes fell into the category ‘biological process’ and ‘molecular function’ respectively. In the comparison between Z01172R vs Z0110R, 2 and 8 GO classes fell into the category ‘biological process’ and ‘molecular function’ respectively. In Z011R, 5 GO classes most relevant to the salt-stress response were identified within its three comparisons: ‘response to stress’, ‘response to oxidative stress’, ‘antioxidant activity’, ‘peroxidase activity’ and ‘oxidoreductase activity, acting on peroxide as acceptor’ (Online Resource 3). These results showed that Z011 responded to salt stress faster than did Z004. The stress response occurred after 1 h of NaCl treatment. When the GO classes most relevant to salt stress in Z004R and Z011R were compared, it was found that the 5 GO classes in Z011R coincided with those in Z004R. However, ‘extracellular region’, ‘oxidoreductase activity, acting on paired donors, with incorporation or reduction of molecular oxygen’, ‘iron ion binding’, ‘ubiquitin-protein transferase activity’, ‘ubiquitin-like protein transferase activity’ and ‘sequence-specific DNA binding’ were unique to Z004R and might be related to the differences in salt tolerance between Z004 and Z011.

In the Z0110R vs Z0040R comparison, the category ‘biological process’ had 1 GO class and ‘molecular function’ had 8 GO classes. In the Z0111R vs Z0041R comparison, the category ‘biological process’ had 2 GO classes, ‘cellular component’ had 1 GO class and ‘molecular function’ had 22 GO classes. In the Z01124R vs Z00424R comparison, the category ‘biological process’ had 11 GO classes and ‘molecular function’ had 8 GO classes. In the Z01172R vs Z00472R comparison, the category ‘biological process’ had 2 GO classes and ‘molecular function’ had 8 GO classes (Online Resource 3). Comparing with 0 h, the mutual GO class of Z011R vs Z004R comparisons among 1 h, 24 h and 72 h was ‘response to stress’. These results showed Z011 had faster and stronger stress response in salt tolerance.

### KEGG pathway analysis of DEGs in the Z004 and Z011 roots

The DEGs in the Z004 and Z011 roots were mapped to KEGG pathways of *Oryza sativa*. In the Z0041R vs Z0040R, Z00424R vs Z0040R and Z00472R vs Z0040R comparisons, 40, 67 and 72 DEGs, respectively, involving 23, 40 and 32 pathways, respectively, were assigned to KEGG pathways (Online Resource 4). In the Z0111R vs Z0110R, Z01124R vs Z0110R and Z01172R vs Z0110R comparisons, 100, 202 and 39 DEGs, respectively, involving 41, 52 and 26 pathways, respectively, were assigned to KEGG pathways (Online Resource 4). In the Z0110R vs Z0040R, Z0111R vs Z0041R, Z01124R vs Z00424R and Z01172R vs Z00472R comparisons, 266, 114, 54 and 119 DEGs, respectively, involving 61, 44, 36 and 52 pathways, respectively, were assigned to KEGG pathways (Online Resource 4). The major pathways identified in the above comparisons were ‘metabolic pathways’, ‘biosynthesis of secondary metabolites’, ‘plant hormone signal transduction’, ‘MAPK signalling pathway – plant’ and ‘phenylpropanoid biosynthesis’.

**Fig. 4:**
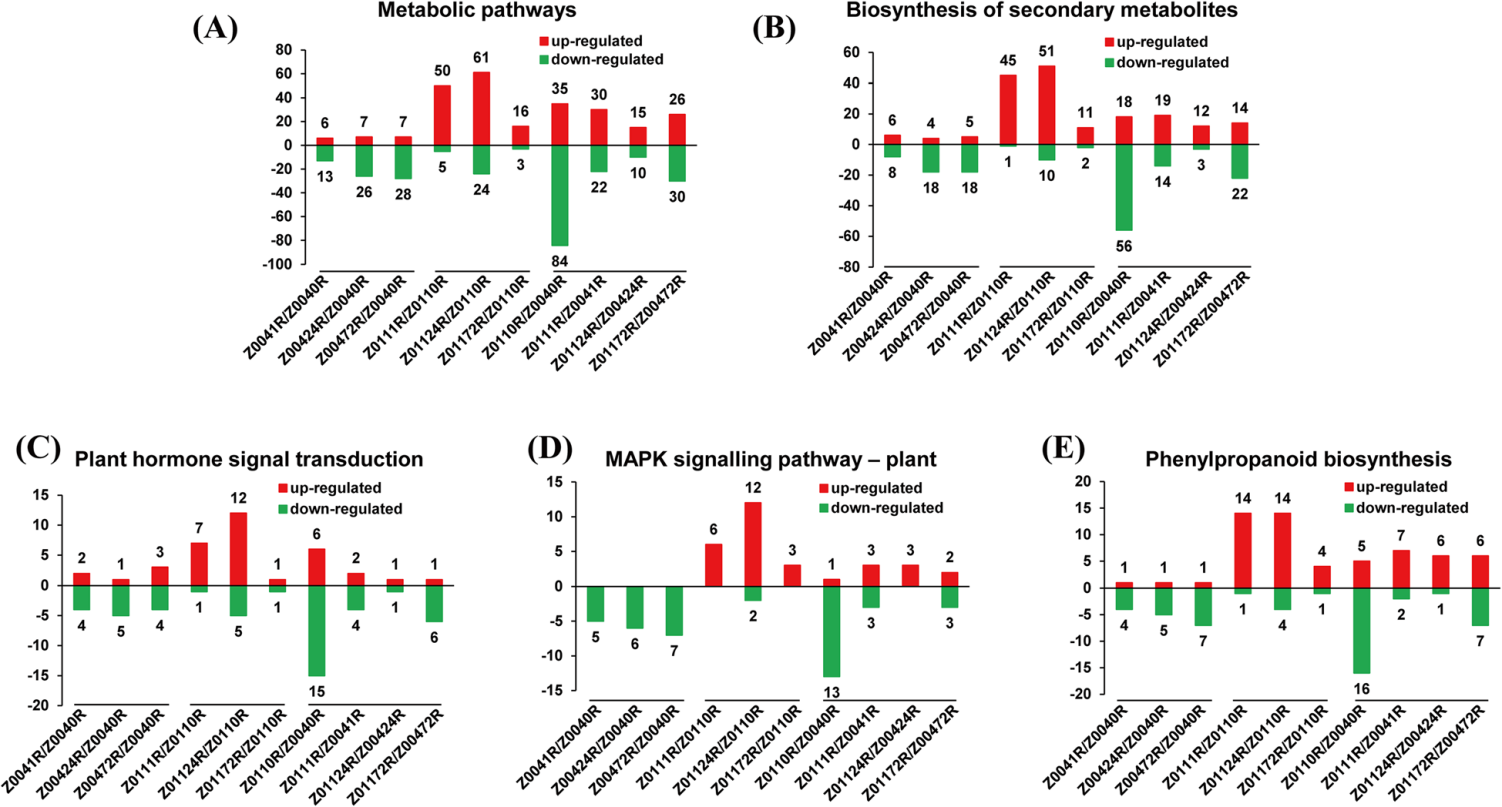
Number of up- or downregulated DEGs enriched in five KEGG pathways in Z004 roots and Z011 roots. **a** The number of up- or downregulated DEGs enriched in metabolic pathways in Z0041R vs Z0040R, Z00424R vs Z0040R, Z00472R vs Z0040R, Z0111R vs Z0110R, Z01124R vs Z0110R, Z01172R vs Z0110R, Z0110R vs Z0040R, Z0111R vs Z0041R, Z01124R vs Z00424R and Z01172R vs Z00472R. **b** The number of up- or downregulated DEGs enriched in the biosynthesis of secondary metabolites in Z0041R vs Z0040R, Z00424R vs Z0040R, Z00472R vs Z0040R, Z0111R vs Z0110R, Z01124R vs Z0110R, Z01172R vs Z0110R, Z0110R vs Z0040R, Z0111R vs Z0041R, Z01124R vs Z00424R and Z01172R vs Z00472R. **c** The number of up- or downregulated DEGs enriched in plant hormone signal transduction in Z0041R vs Z0040R, Z00424R vs Z0040R, Z00472R vs Z0040R, Z0111R vs Z0110R, Z01124R vs Z0110R, Z01172R vs Z0110R, Z0110R vs Z0040R, Z0111R vs Z0041R, Z01124R vs Z00424R and Z01172R vs Z00472R. **d** The number of up- or downregulated DEGs enriched in the MAPK signaling pathway - plant in Z0041R vs Z0040R, Z00424R vs Z0040R, Z00472R vs Z0040R, Z0111R vs Z0110R, Z01124R vs Z0110R, Z01172R vs Z0110R, Z0110R vs Z0040R, Z0111R vs Z0041R, Z01124R vs Z00424R and Z01172R vs Z00472R. **e** The number of up- or downregulated DEGs enriched in phenylpropanoid biosynthesis in Z0041R vs Z0040R, Z00424R vs Z0040R, Z00472R vs Z0040R, Z0111R vs Z0110R, Z01124R vs Z0110R, Z01172R vs Z0110R, Z0110R vs Z0040R, Z0111R vs Z0041R, Z01124R vs Z00424R and Z01172R vs Z00472R

Statistical analysis of the number of DEGs in the above five pathways revealed that the expression of most in Z004 was downregulated, while the expression of most in Z011 was upregulated (Fig. 4). Especially in the MAPK signalling pathway, which is closely related to the plant response to abiotic stress, the expression of all DEGs in Z004 was downregulated; however, in Z011, there were only 2 DEGs whose expression was downregulated, whereas that of the all other DEGs was upregulated (Fig. 4d). In Z011, the number of DEGs was greatest after 24 h of NaCl treatment, and the number of DEGs was lowest after 72 h (Fig. 4). The comparisons of Z011 and Z004 in different time points performed that the number of downregulated DEGs was decreased obviously after the salt treatment (Fig. 4). In general, the number of DEGs involved in the salt-stress response in Z011 was significantly greater than that in Z004, and the number genes whose expression was upregulated obviously increased.

### Identification of DEGs from comparisons of the Z004 and Z011 roots

**Fig. 5:**
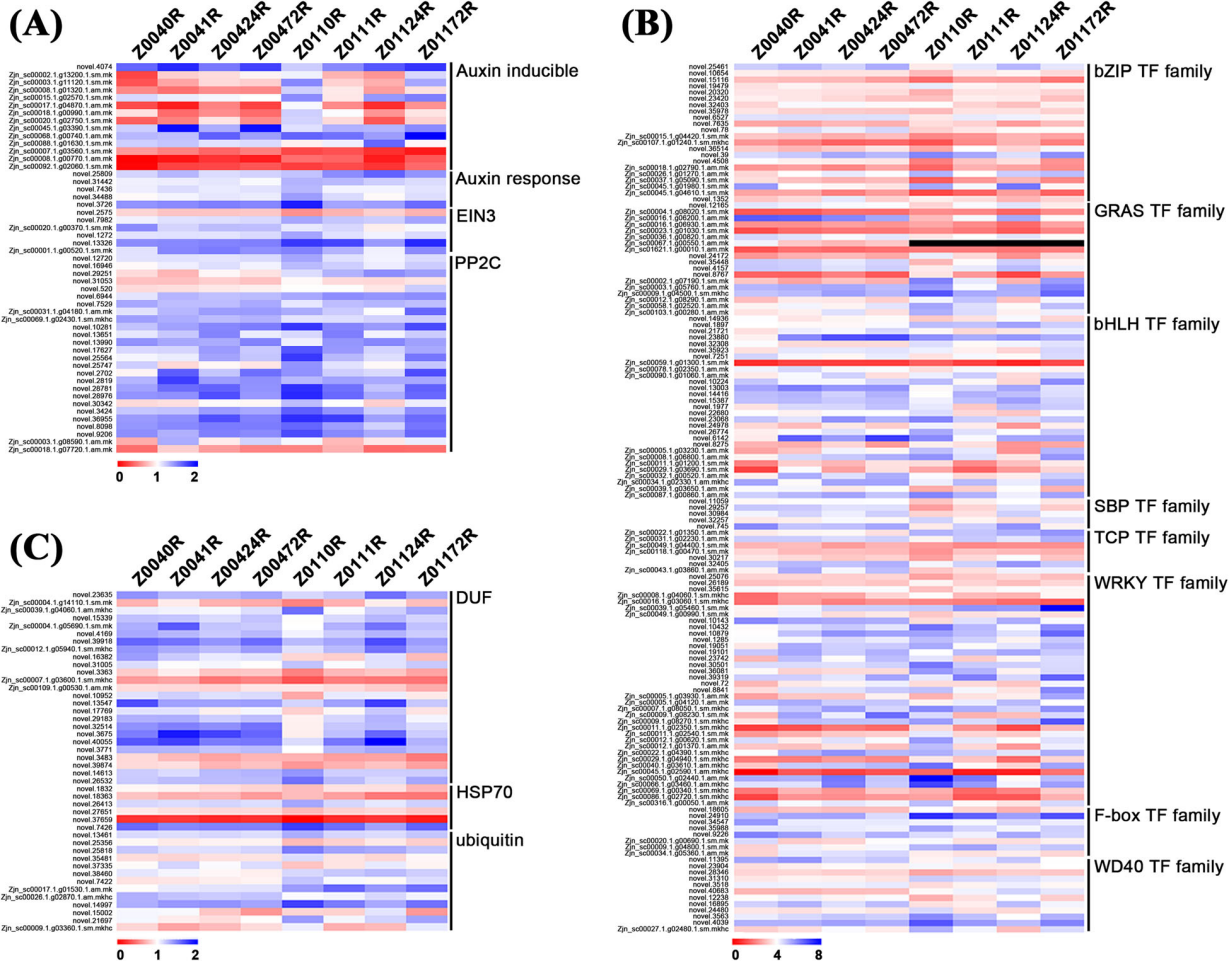
Heatmap of 233 DEGs in the roots of Z004 and Z011 at four time points after salt treatments. The 233 DEGs were grouped into 3 main categories, and the red and blue rectangles represent the scale of the expression levels of each gene (log_2_FPKM). The red rectangles represent upregulated genes, and blue rectangles represent the downregulated genes

To narrow the selection range of the DEGs, we focused on those within plant hormone signal transduction families, TF families and other gene families that have been reported to be involved in salt tolerance. A total of 233 DEGs were identified with a |log2(fold change)| ≥ 2 and padj < 0.05 in any one comparison and selected from six different comparisons of Z004 and Z011 roots. With respect to the hormone pathways, the ABA signalling pathway had the most DEGs (26 genes), followed by the auxin signal pathway (19 genes) (Fig. 5a, Online Resource 5). The DEGs in the ABA signalling pathway all belonged to the *protein phosphatase 2C* (*PP2C*) family; however, in the auxin signalling pathway, 14 DEGs were related to auxin induction, and 5 DEGs were related to the auxin response (Fig. 5a, Online Resource 5). In addition, 6 DEGs in the ethylene signalling pathway belonged to the *ethylene insensitive 3* (*EIN3*) family (Fig. 5a, Online Resource 5). With respect to TFs, a total of 8 TF families were identified. The *WRKY* TF family had the most DEGs (37 genes), followed by the *bHLH* TF family (29 genes) (Fig. 5b, Online Resource 5). In addition, members of the *bZIP* TF family (22 genes), *GRAS* TF family (18 genes), *WD40* TF family (12 genes), *F-box* TF family (8 genes), *TCP* TF family (7 genes) and *SBP* TF family (5 genes) were identified (Fig. 5b, Online Resource 5). Moreover, a total of 25 genes in the DUF family, 13 ubiquitin genes and 6 heat-shock protein 70 (HSP70) gene were also identified (Fig. 5c, Online Resource 5).

From the above 233 DEGs, we selected 44 whose expression was significantly contrastingly up- or downregulated between the Z004 and Z011 roots after salt treatment (Table 1). The expression of seven DEGs in the hormone signalling pathway was downregulated in the Z004 roots and upregulated in the Z011 roots. Among these DEGs, three belonged to the auxin-responsive protein *small auxin-up RNA* (*SAUR*) family in the auxin signalling pathway, one belonged to the auxin response factor family in the auxin signaling pathway, one DEG belonged to the *EIN3* family in the ethylene signalling pathway, and two DEGs belonged to the *PP2C* family in the ABA signalling pathway. Six of these DEGs in the Z011 roots had the highest FPKM values at 24 h after salt treatment except *novel.25809*, which suggested that these six genes might have significant effects on salt tolerance (Table 1). In our research, members of various TF families involved in salt tolerance were identified. The major TF families identified were the *WRKY* and *bHLH* families. Twelve *WRKY* and nine *bHLH* TFs are listed in Table 1. The expression of all the *WRKY* was downregulated in the Z004 roots and upregulated in the Z011 roots. Moreover, with the exception of that of *Zjn_sc00039.1.g03650.1.am.mk* (*bHLH30*), the expression of 8 *bHLH* TFs was downregulated in the Z004 roots and 7 of them upregulated in the Z011 roots. In the *bZIP* TF family, six DEGs were selected, three of whose expression was upregulated in the Z004 roots and downregulated in the Z011 roots; and *novel.1352* (*bZIP53*) had the opposite tendency of expression. In addition, the expression of three *GRAS* TF family members, two *WD40* TF family members and one *SBP* TF family member was downregulated in the Z004 roots and upregulated in the Z011 roots, except *Zjn_sc00016.1.g06200.1.am.mk* (*SCL9*). The expression of the *TCP* TF family member *novel.30217* (*TCP7*) was upregulated in the Z004 roots and downregulated in Z011 roots (Table 1), and two *DUF* family members, *Zjn_sc00012.1.g05940.1.sm*.*mkhc* (*DUF315*) and *novel.26532* (*DUF1671*), were also selected and displayed a contrasting expression trend, which might indicate that they have opposite functions (Table 1). From the above, the expression of 27 DEGs was downregulated in the Z004 roots and upregulated in the Z011 roots, and that of 4 DEGs was upregulated in the Z004 roots and downregulated in the Z011 roots, indicating that these genes might be related to the salt tolerance of zoysiagrass. Among them, the number of upregulated genes in the roots of the salt-tolerant accession Z011 roots was significantly greater than the number of downregulated genes.

**Table 1:**
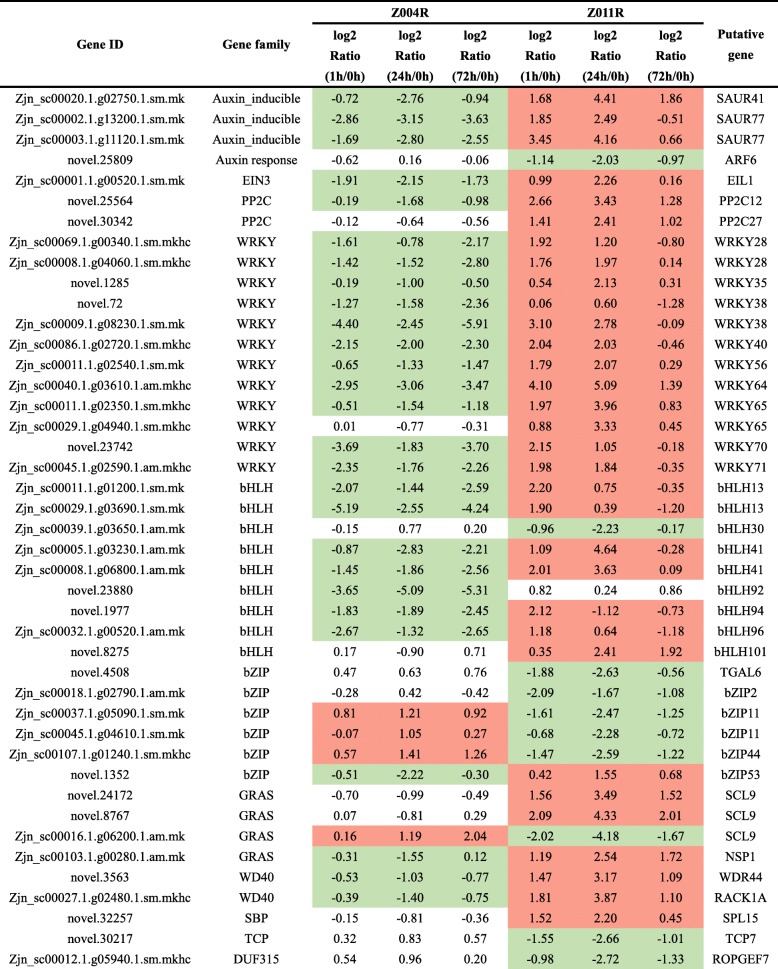
Selected up- (red) or down-regulated (green) DEGs in Z004 and Z011 roots after salt treatments

### Verification of the transcriptome sequencing data of the Z004 and Z011 roots

To verify the reliability of the transcriptome sequencing data of the Z004 and Z011 roots, twenty of the 39 DEGs whose expression was significantly contrastingly up- and downregulated signal pathway in the Z004 and Z011 roots after salt treatment were selected and validated via quantitative real-time PCR (qRT-PCR). With the exception of *Zjn_sc00029.1.g04940.1.sm*.*mkhc* and *Zjn_sc00011.1.g02540.1.sm.mk*, approximately 90.0% of the DEGs were consistent between the RNA-seq and qRT-PCR data (Supplementary Figure S2). The results of the qRT-PCR and RNA-seq data were generally consistent, indicating that our transcriptome sequencing data of the Z004 and Z011 roots were reliable.

## Discussion

### Phenotypic and physiological responses to salt stress in *Z. japonica* Steud

*Zoysia* is a warm-season turfgrass that is widely used in home lawns, football fields and ecological management [50]. Zoysiagrass is recognized for its salt tolerance, hardiness, and drought tolerance and is suitable as a high-quality salt-tolerant turfgrass for landscaping in areas with saline soils [10]. Previous studies have shown that the salt tolerance of *Zoysia* is negatively correlated with the content of Na^+^ and positively correlated with the content of K^+^ in leaf fluids [25]. Salt-tolerant plants materials have a strong ability to maintain the K^+^/Na^+^ ratio in their leaves and roots [25, 33, 34]. Our data from two accessions the (salt-sensitive material Z004 and salt-tolerant material Z011) with contrasting salt tolerances support these previous studies. After 350 mM NaCl treatment for 40 days, Z011 was more salt tolerant than was Z004, grew better, and had greater biomass (Fig. 1a-e). Compared with those of Z004, the Na^+^ concentrations in the leaves and secretions of Z011 were maintained at significantly lower levels, but there were no differences in the roots (Fig. 2a-c). Moreover, there was no difference in K^+^ concentration in the leaves of Z011 compared with Z004; however the K^+^ concentration was significantly greater in the the roots of Z011, and the K^+^ secretion was also lower in the roots of Z011 (Fig. 2d-f). These results indicated that, compared with Z004, Z011 may have improved salt tolerance by reducing the transport of Na^+^ from the roots to the leaves, increasing the absorption of K^+^ in the roots and reducing the secretion of K^+^ from leaves to maintain a significantly greater K^+^/Na^+^ ratio (Fig. 2g, h).

### Transcriptome sequencing of Z004 and Z011 and DEG identification in response to salt stress

Xie et al. [58] presented the first comprehensive transcriptome data of *Z. japonica* Steud. roots after 30 min of NaCl treatment, and a total of 32,849 unigenes and 4842 SSRs were identified. However, the important regulatory capabilities of the roots and leaves of this species under salt stress, and the key time point for salt tolerance regulation remain unknown. Our research involved the sampling of leaves and roots from Z004 and Z011 at 0 h, 1 h, 24 h and 72 h after treatment with 350 mM NaCl, and a total of 59,271 unigenes and 29,675 novel genes were revealed by RNA-seq. PCA revealed that the Z011 root samples had the highest dispersion degree, and the 24-h samples of the roots of both Z004 and Z011 were separated from those of other samples (Fig. 3a). Moreover, the number of DEGs of Z011 roots was obviously more than that in the Z004 roots, and the number of DEGs at 24 h (Z01124R vs Z0110R) was 2.28 and 5.64 times that at 1 h (Z0111R vs Z0110R) and 72 h (Z01172R vs Z0110R) (Fig. 3b, c, Supplementary Figure S1). Comparing the Z011 and Z004, the number of DEGs of roots was obviously more than leaves (Fig. 3b). These results indicated that the roots might make a significant contribution to the difference in salt tolerance between Z004 and Z011 and the 24 h might be a relatively important time point for the salt-stress response.

### Plant hormone signal transduction is involved in salt tolerance

Further evidence from the KEGG pathway enrichment analysis of the DEGs in the Z004 and Z011 roots demonstrated that plant hormone signal transduction has important effects on salt tolerance (Fig. 4c). Seven DEGs involved in plant hormone signal transduction were selected, and the expression of six of them was downregulated in the Z004 roots and upregulated in the Z011 roots. Among these DEGs, one *SAUR41* and two *SAUR77* genes belonged to the auxin-responsive protein *SAUR* family in the auxin signalling pathway, one *ARF6* gene belonged to the auxin response factor (ARF) family in the auxin signaling pathway, one *ethylene-insensitive-3-like-1* (*EIL1*) gene belonged to the *EIN3* family in the ethylene signalling pathway and one *PP2C12* and one *PP2C27* gene each belonged to the *PP2C* family in the ABA signalling pathway (Table 1).

*SAURs* compose a family of auxin-responsive genes that play an important role in the regulation of plant growth and development. However, the function of members of the *SAUR* family in terms of salt tolerance has rarely been reported. Previous studies have shown only that the expression of *TaSAUR75* is downregulated in wheat roots after salt stress, and increased root length, survival rate and expression of some salt and drought stress-responsive genes were detected in *TaSAUR75*-overexpressing transgenic plants compared with CK plants [13]. In *Arabidopsis*, overexpression of *AtSAUR41* promotes hypocotyl elongation and increases both primary root growth and lateral root number [19]. Yeast two-hybrid experiments showed that *AtSAUR77* might participate in ethylene receptor signalling and promote plant growth [27]. However, the function of these two genes in salt tolerance has not yet been reported. In our research, under salt tolerance, the expression of both *ZmSAUR41* and *ZmSAUR77* was downregulated in the Z004 roots and upregulated in the Z011 roots, indicating that *ZmSAUR41* and *ZmSAUR77* might improve salt tolerance (Table 1). ARF family bind to promoters of many auxin-regulated genes with auxin response elements (AuxREs, 5′ tgtctc 3′) and regulate the expression of auxin-induced genes [14, 29]. In *Arabidopsis*, *ARF6* and *ARF8* are the target genes of miR167, and miR167 is induced by salinity and drought [20]. In our research, the expression of *ZmARF6* was downregulated in the Z011 roots after salt treatments, indicating that *ZmARF6* might negative regulated salt tolerance (Table 1).

Ethylene considered a stress hormone involved in the plant response to salt tolerance. *EIN3* and *EIL1* are two ethylene-activated TFs and have been reported to be important for improving salt tolerance. In *Arabidopsis*, accumulation of EIN3/EIL1 is induced by high salinity and likely enhances reactive oxygen species (ROS) scavenging at the seedling stage to promote salinity tolerance, as compared with wild-type plants, *ein3 eil1* double mutants have greater levels of ROS and lower levels of peroxidase-encoding transcripts and are hypersensitive to salt tolerance [38]. However, in contrast, overexpression *OsEIL1* and *OsEIL2* in rice resulted in salt hypersensitivity at the seedling stage, and the lack of *OsEIL1* and *OsEIL2* functionality increased salt tolerance. Furthermore, the negative regulation of *OsEIL1* and *OsEIL2* in salt tolerance might contribute to the regulation of *high-affinity K*^*+*^*transporter 2;1* expression and the absorption of Na^+^ in roots [60]. In our research, under salt tolerance, the expression of *ZmEIL1* was downregulated in Z004 roots and upregulated in Z011 roots, indicating that *ZmEIL1* might have a positive effect on salt tolerance, which is consistent with the function of *AtEIL1* in *Arabidopsis* (Table 1).

*PP2C* enzymes are key elements involved in the ABA signalling pathway. In the present study, the expression levels of *PP2C* family genes differed in response to salt tolerance. Overexpressing *AtPP2CG1* (*A. thaliana protein phosphatase 2C G Group 1*) in *Arabidopsis* can enhance salt tolerance, whereas a lack of *AtPP2CG1* function reduces salt tolerance. In addition, *AtPP2CG1* upregulated the expression levels of some stress-responsive genes under salt treatment, including *RD29A*, *RD29B*, *DREB2A* and *KIN1* [31]. In maize, salt treatment induced intron methylation of *ZmPP2C* and then significantly downregulated the expression of *ZmPP2C* [47]. Transcriptomic analysis of *Camellia sinensis* revealed that multiple *PP2C* members participate in the salt tolerance response, such as *PP2C2*, *PP2C3*, *PP2C14*, *PP2C51* and *PP2C60,* whose expression was upregulated under salt treatment; however, the expression of *PP2C12*, *PP2C27* and *PP2C54* was downregulated [52]. In our study, after salt treatment, the expression of both *ZmPP2C12* and *ZmPP2C27* was downregulated in the Z004 roots and upregulated in the Z011 roots, indicating that *ZmPP2C12* and *ZmPP2C27* might be positive regulators of salt tolerance (Table 1).

### TFs involved in salt tolerance

In our research, members of various TF families involved in salt tolerance were identified. Among them, the members of the *WRKY* and *bHLH* TF families were the most common (Table 1). The *WRKY* TF family is known to be involved in various physiological processes and many aspects of the plant defence system. We identified twelve differentially expressed *WRKY* genes, and eight of them were reported to be related to salt tolerance. In *Gossypium raimondii*, Cai et al. [3] reported that *WRKY35*, *WRKY40* and *WRKY64* were induced by salt, and the expression levels were significantly upregulated. Moreover, *AtWRKY40* was found to be induced in response to salt stress in *Arabidopsis* [42]. Overexpressing *FcWRKY40* of *Fortunella crassifolia* in tobacco and lemon increased salt tolerance, while silencing *FcWRKY40* decreased salt tolerance [8]. Wang et al. [55] cloned the *WRKY56* gene from *Populus simonii* × *Populus nigra*, which was significantly induced by salt treatment, and transformed it into *Arabidopsis*; the resulting *WRKY56-*overexpressing transgenic *Arabidopsis* plants were more salt tolerant than were the wild-type plants, and the fresh weight and germination of the latter increased [55]. A *PsnWRKY70* gene was also cloned from *P. simonii* × *P. nigra* and confirmed the response to salt stress in *PsnWRKY70*-repressed plants, which exhibited enhanced salt tolerance [64]. In addition, AtWRKY70 has been reported to regulate salt stress by interacting with the Cys2/His2 zinc finger protein Zat7 [7]. *WRKY71* and *WRKY28* are homologues and are induced by high salinity. Overexpression of *WRKY71* or *WRKY28* resulted in insensitive flowering of *Arabidopsis* plants in response to high salinity, while downregulated of *WRKY71* and *WRKY28* resulted in more sensitive flowering of *Arabidopsis* [62]. Given that the expressions of *WRKYs* in our research were mainly downregulated in the Z004 roots and upregulated in Z011 roots, *WRKY* genes may play positive roles in salt tolerance.

The *bHLH* TF family has been reported to participate in the regulation of abiotic stress-related signal transduction. Nine differentially expressed *bHLH* genes were identified in our research, and five of them have been reported to be regulated by NaCl (Table 1). The *bHLH30* genes in chrysanthemum, evergreen tree and upland cotton are induced under salt stress and have been reported to increase salt tolerance in yeast [6, 45, 56]. In *Arabidopsis*, two presumptive paralogues of *bHLH92*, *bHLH41* and *bHLH42,* are induced by salt treatment [16]. Moreover, transcriptome analysis of the salinity tolerance of *Brassica juncea* revealed that the expression of *bHLH101* was downregulated under salt treatment [44]. In our research, the expressions of the *bHLH13*, *bHLH41*, *bHLH92*, *bHLH94*, *bHLH96* and *bHLH101* genes were downregulated in the Z004 roots and upregulated or maintained in the Z011 roots, indicating that these *bHLH* genes might be positive regulators of salt tolerance. Among them, the expression trend of *bHLH101* in *Zoysia* was opposite that in *B. juncea* [44], indicating that *bHLH101* may have different functions in different species. In addition, *bHLH30*, whose expression was upregulated in the Z004 roots and downregulated in the Z011 roots, might play a negative role in the salt tolerance of *Zoysia,* which contrasts with the results of previous reports.

Members of the *bZIP* TF family have important roles in many biological processes, and some *bZIP* TF family members have been reported to exert biological functions under salt stress. Transgenic plants overexpressing *AtbZIP1*, *ZmbZIP72*, *GmbZIP132* and *ZmABP9* presented enhanced tolerance to salt stress [28, 46, 53, 61]. Six differentially expressed *bZIP* genes were identified in our research, and *bZIP2*, *bZIP44* and *bZIP53* were reported to be involved in salt tolerance (Table 1). The transcript level of the *LebZIP2* gene in *Lycopersicon esculentum* increased after salt-stress treatments, and the *bZIP1* and *bZIP53* mutants displayed reduced salt tolerance [15, 43]. Overexpressing *GmbZIP44* gene could increase the salt tolerance of transgenic plants in soybean [28]. In our research, the expression of *TGAL6*, *bZIP2*, *bZIP11* and *bZIP44* was upregulated in the Z004 roots and downregulated in the Z011 roots, while the expression of *bZIP53* was downregulated in the Z004 roots and upregulated in the Z011 roots, indicating that different bZIP members might play different roles in the salt tolerance process in *Z. japonica*.

Among the remaining seven TFs (Table 1), only the salt tolerance function of the *RACK1A* gene has been clearly elucidated. In rice, suppression of *OsRACK1A* increased salt tolerance by maintaining low Na^+^ and high K^+^ concentrations in both the roots and leaves [63]. Our data showed that the expression of three *GRAS* TF family members (*SCL9* and *NSP1*), two *WD40* TF family members (*WDR44* and *RACK1A*) and one *SBP* TF family member (*SPL15*) was downregulated in the Z004 roots and upregulated in the Z011 roots, while the expression of one *GRAS* TF family member (*SCL9*) and one *TCP* TF family member (*TCP7*) was upregulated in the Z004 roots and downregulated in the Z011 roots. Thus, these genes may have important roles in salt tolerance, but their functions require additional research.

### The *DUF* family is involved in the salt tolerance of *Z. japonica*

DUF family members compose a large number of uncharacterized protein families within the Pfam database (http://pfam.xfam.org/family), which contains approximately 3000 families [1]. Some DUF proteins are active in plant development [2, 4], and other members of *DUF* families are involved in the stress response [18, 54], especially the salt-stress response. The *AhDGR2* gene in *Amaranthus hypochondriacus* encodes a DUF642 domain-containing protein, and plants overexpressing *AhDGR2* present increased sensitivity to NaCl treatment [37]. *OsDSR2*, which encodes a DUF966 domain-containing protein, also negatively regulates salt stress in rice [32]. However, overexpressing *SIDP361* (a DUF1644 protein-coding gene) in rice significantly enhances salt tolerance at both the seedling and heading stages [24]. The expression of *OsDUF810.7* significantly increases under salt treatment, and overexpression of this gene in *E. coli* improves the salt tolerance of the bacterium [23]. Two *DUF* family members, *ROPGEF7* (a DUF 315 protein-coding gene) and *UFSP* (a DUF 1671 protein-coding gene), were selected in our research and showed an inverse expression trend (Table 1), indicating that these two DUF genes might play important roles in the salt tolerance process in *Z. japonica* and might have contrasting functions.

## Conclusions

Our research performed that salt-tolerant *Z. japonica* accession Z011 may have improved salt tolerance by reducing Na^+^ transport from the roots to the leaves, increasing K^+^ absorption in the roots and reducing K^+^ secretion from the leaves to maintain a significantly greater K^+^/Na^+^ ratio. Twenty-four hours might be a relatively important time point for the salt-stress response of zoysiagrass. The auxin signal transduction family, ABA signal transduction family, *WRKY* TF family and *bHLH* TF family may be the most important families in *Zoysia* salt-stress regulation. This study provides fundamental information concerning the salt-stress response of *Zoysia* and improves the understanding of molecular mechanisms in salt-tolerant plants.

## Methods

### Plant materials and treatment

On the basis of the salt tolerance of 206 zoysiagrass accessions identified by their leaf firing, relative shoot clipping dry weight, verdure dry weight and root dry weight (H. L. Guo, unpublished data), the salt-sensitive *Z. japonica* accession Z004 and the salt-tolerant *Z. japonica* accession Z011 were used in this study. Both accessions were collected in 1995 by Jianxiu Liu, turfgrass major of Institute of Botany, Jiangsu Province and Chinese Academy of Sciences, China. Z004 was collected in a wild grassland in Lushan area (Jiangxi, China, 28°36′N, 116°00′E) and Z011 was collected in a hilly land of Lanxi area (Zhejiang, China, 29°13′N, 119°30′E). As *Zoysia* Willd. is not endangered, collection of samples for scientific purposes was permitted by local legislation. Professor Shouliang Chen, taxonomy major, and Jianxiu Liu, turfgrass major of Institute of Botany, Jiangsu Province and Chinese Academy of Sciences, undertook the formal identification of the samples according to *flora of Reipublicae Popularis Sinicae* (Vol.10, No.1, 1990). Previous morphological and DNA analyses also confirmed the correct identification of the two accessions [12, 30]. Both accessions were maintained at an experimental field of the Institute of Botany, Jiangsu Province and Chinese Academy of Sciences. Twenty uniform sprigs of both materials were transplanted from the experimental field of the Institute of Botany were planted into 9-cm-diameter and 6-cm-deep plastic pots filled with coarse silica sand. Ten pots were planted per treatment and material. The pots were suspended over tanks (66.56 × 45.56 × 17.0 cm^3^) filled with 45 L of 1/2-strength Hoagland’s solution. The grasses were clipped weekly until growth was consistent and were cultivated in a greenhouse with a day/night temperature of 35/28 °C, 16 h of light/8 h of dark, 75% relative humidity and 800 μmol m^− 2^ s^− 1^ of photosynthetically active radiation. NaCl treatment (350 mM) was initiated after 2 months of cultivation.

### Observations and measurements of the salt tolerance of *Z. japonica* Steud

After the turfgrasses were subjected to salt treatment for 40 days, the leaf firing was assessed by visual rating via a scale of 1 (slight firing) to 9 (severe firing). All the treated grasses and CK grasses were divided into three parts: shoot clippings, verdure and roots. Among them, the part of the grass blades clipped to a height of 4.0 cm were considered shoot clippings, and the part excluding the shoot clippings and roots was considered the verdure. These three parts were dried at 70 °C for 48 h, after which the dry weight was determined. The relative dry weights were then calculated according to the following equation: relative dry weight = Wt/Wo × 100%, where Wt is the dry weight in the NaCl group, and Wo is the dry weight in the CK group. Three biological replicates were tested, and data from individual measurements were averaged and analysed via SPSS statistical software 18.0 (*t* test) (SPSS Inc., Chicago, IL, USA).

### Determination of the concentrations of Na^+^ and K^+^

To determine the concentrations of Na^+^ and K^+^ in the leaves and roots, 20 mg of dry powder samples was placed into sealed test tubes that contained 15 ml of ddH_2_O. All the samples were boiled for 1 h and incubated at room temperature for 24 h. After the samples were filtered, the extracted Na^+^ and K^+^ concentrations were measured by flame photometry (Model FP6410; Shanghai Xinyi Instruments Inc., Shanghai, China) and calculated on the basis of the tissue dry weight (mmol kg^− 1^). To determine the secretion of Na^+^ and K^+^, the leaves were washed with ddH_2_O 3 times after 5 days of salt treatment to remove the salt crystals secreted from the leaf surface. Seven days later, 4–8 pieces of mature leaves were removed and placed into centrifuge tubes. Each centrifuge tube was supplemented with 10 ml of ddH_2_O and shaken for 5 s to fully dissolve the salt crystals on the leaf surface into the ddH_2_O. Afterward, the leaves in the centrifuge tubes were removed and dried at 70 °C for 48 h, after which the dry weight was determined. The Na^+^ and K^+^ concentrations within 10 ml of ddH_2_O were measured by flame photometry and calculated on the basis of the corresponding leaf dry weight (mmol kg^− 1^). The K^+^/Na^+^ ratio was considered the ratio of the K^+^ concentration to the Na^+^ concentration. Three biological replicates were analysed, and the data from the individual measurements were averaged and analysed using SPSS statistical software 18.0 (*t* test) (SPSS Inc., Chicago, IL, USA).

### RNA-seq

The leaf and root tissues of Z004 and Z011 at four time points (0 h, 1 h, 24 h, and 72 h) after salt treatment were sampled, frozen in liquid nitrogen and stored at − 80 °C. Each collected sample was 0.1 g, and three biological replicates were analysed. The total RNA was extracted [58], and every three biological replicate RNA samples were mixed fully into one RNA library. A total of 16 RNA libraries were generated after the samples were pooled: Z0040L (Z004, 0 h, leaf), Z0041L (Z004, 1 h, leaf), Z00424L (Z004, 24 h, leaf), Z00472L (Z004, 72 h, leaf), Z0040R (Z004, 0 h, root), Z0041R (Z004, 1 h, root), Z00424R (Z004, 24 h, root), Z00472R (Z004, 72 h, root), Z0110L (Z011, 0 h, leaf), Z0111L (Z011, 1 h, leaf), Z01124L (Z011, 24 h, leaf), Z01172L (Z011, 72 h, leaf), Z0110R (Z011, 0 h, root), Z0111R (Z011, 1 h, root), Z01124R (Z011, 24 h, root), and Z01172R (Z011, 72 h, root). The cDNA library construction and transcriptome sequencing were performed by Novogene (Tianjin, China) (http://www.novogene.com/) on an Illumina HiSeq™ 2000 platform, and 125 bp/150 bp paired-end reads were generated. The datasets are available in the NCBI repository http://www.ncbi.nlm.nih.gov/bioproject/PRJNA559944.

### De novo assembly, gene expression levels and identification of DEGs

Clean reads were obtained from the raw data by the removal of reads containing adapters, reads with ambiguous ‘N’ bases and reads of low quality. The Q20, Q30 and GC content of the clean reads were calculated, and the subsequent analyses were based on the high-quality clean reads. De novo assembly of the *Z. japonica* transcriptome from the reference genome of zoysiagrass [48] was accomplished via Hisat2 (v2.0.5) [17]. The mapped reads of each sample were then assembled, and the novel transcripts were predicted by StringTie (v1.3.3b) [39]. The gene expression levels were calculated according to the FPKM method [36]. The edgeR R package (3.18.1) [41] was subsequently used to analyse significant differences in expression with padj < 0.05 and fold change > 2, and DEGs were identified with a |log2(fold change)| > 1 and padj < 0.05.

### GO and KEGG enrichment analyses of DEGs

All the DEGs were mapped to terms identified from the GO and KEGG (http://www.genome.jp/kegg/) enrichment analyses, and the clusterProfiler R package was used to analyse the GO enrichment of the DEGs and to test the statistical enrichment of the DEGs in the KEGG pathways. The GO terms and KEGG categories with padj < 0.05 (corrected *P*-value) were selected as significant GO classifications and KEGG pathways for the DEGs in each comparison.

### qRT-PCR validation of transcription

Twenty DEGs were selected from the 44 DEGs whose expression was significantly contrastingly up- or downregulated in the Z004 and Z011 roots after salt treatment to validate the reliability of the transcriptome data. The primers of the DEGs were designed using Primer 5.0 software, and *ZjActin* (GenBank: GU290545.1) was used as a housekeeping gene. Each sample consisted of three biological replicates, and the qRT-PCR assays were carried out as described by Xie et al. [58]. The gene primers used are listed in Online Resource 6.

## Supplementary information

**Additional file 1: Figure S1.** Venn diagram of the number of DEGs in the leaves and roots of Z004 and Z011 after salt treatment (a) Venn diagram of the number of DEGs in Z0041L vs Z0040L, Z00424L vs Z0040L, and Z00472L vs Z0040L. (b) Venn diagram of the number of DEGs in Z0041R vs Z0040R, Z00424R vs Z0040R, and Z00472R vs Z0040R. (c) Venn diagram of the number of DEGs in Z0111L vs Z0110L, Z01124L vs Z0110L, and Z01172L vs Z0110L. (d) Venn diagram of the number of DEGs in Z0111R vs Z0110R, Z01124R vs Z0110R, and Z01172R vs Z0110R. **Figure S2.** qRT-PCR validation of 20 genes randomly selected from the 39 DEGs in Table 1 in Z004 and Z011 roots. The error bars indicate the SEs.

**Additional file 2: Online Resource 1** Summary of RNA-seq results and their matches to the *Z. japonica* genome.

**Additional file 3: Online Resource 2** The proportion of reads in the *Z. japonica* genomic exon, intron and intergenic regions.

**Additional file 4: Online Resource 3** Significant GO classification of DEGs in each comparison.

**Additional file 5: Online Resource 4** Pathway classification of the DEGs in each comparison.

**Additional file 6: Online Resource 5** Two hundred twenty-three DEGs selected from each comparison of Z004 and Z011 roots.

**Additional file 7: Online Resource 6** Primer sequences used for qRT-PCR.

**Additional file 8: Online Resource 7** Summary of RNA-seq results and their matches to the rice (*Oryza sativa L. japonica*) genome.

**Additional file 9: Online Resource 8** Summary of RNA-seq results and their matches to the *Sorghum bicolor* genome.

## Data Availability

The sequencing data are available in the NCBI repository http://www.ncbi.nlm.nih.gov/bioproject/PRJNA559944. The datasets analyzed during the current study are available from the corresponding author on reasonable request. All data generated or analyzed during this study are included in this published article [and its Additional files].

## Abbreviations

cDNA: Complementary DNA
CK: Control group
DEGs: Differentially expressed genes
FPKM: Fragments per kb per million fragments
GO: Gene ontology
KEGG: Kyoto Encyclopedia of Genes and Genomes
NCBI: National Center for Biotechnology Information
PCA: Principal component analysis
qRT-PCR: Quantitative real-time PCR
RNA-seq: RNA sequence
TF: Transcription factors
Tr: The treatment group
