# Supplementary material for: Physiological and transcriptomic analyses reveal the mechanisms underlying the salt tolerance of Zoysia japonica Steud
Source: BMC Plant Biol. 2020 Mar 14;20:114. doi: 10.1186/s12870-020-02330-6 (PMC7071773; doi:10.1186/s12870-020-02330-6)
Supplement: Supplementary file 1 — Additional file 1: Figure S1. Venn diagram of the number of DEGs in the leaves and roots of Z004 and Z011 after salt treatment (a) Venn diagram of the number of DEGs in Z0041L vs Z0040L, Z00424L vs Z0040L, and Z00472L vs Z0040L. (b) Venn diagram of the number of DEGs in Z0041R vs Z0040R, Z00424R vs Z0040R, and Z00472R vs Z0040R. (c) Venn diagram of the number of DEGs in Z0111L vs Z0110L, Z01124L vs Z0110L, and Z01172L vs Z0110L. (d) Venn diagram of the number of DEGs in Z0111R vs Z0110R, Z01124R vs Z0110R, and Z01172R vs Z0110R. Figure S2. qRT-PCR validation of 20 genes randomly selected from the 39 DEGs in Table 1 in Z004 and Z011 roots. The error bars indicate the SEs. [file 12870_2020_2330_MOESM1_ESM.pdf]

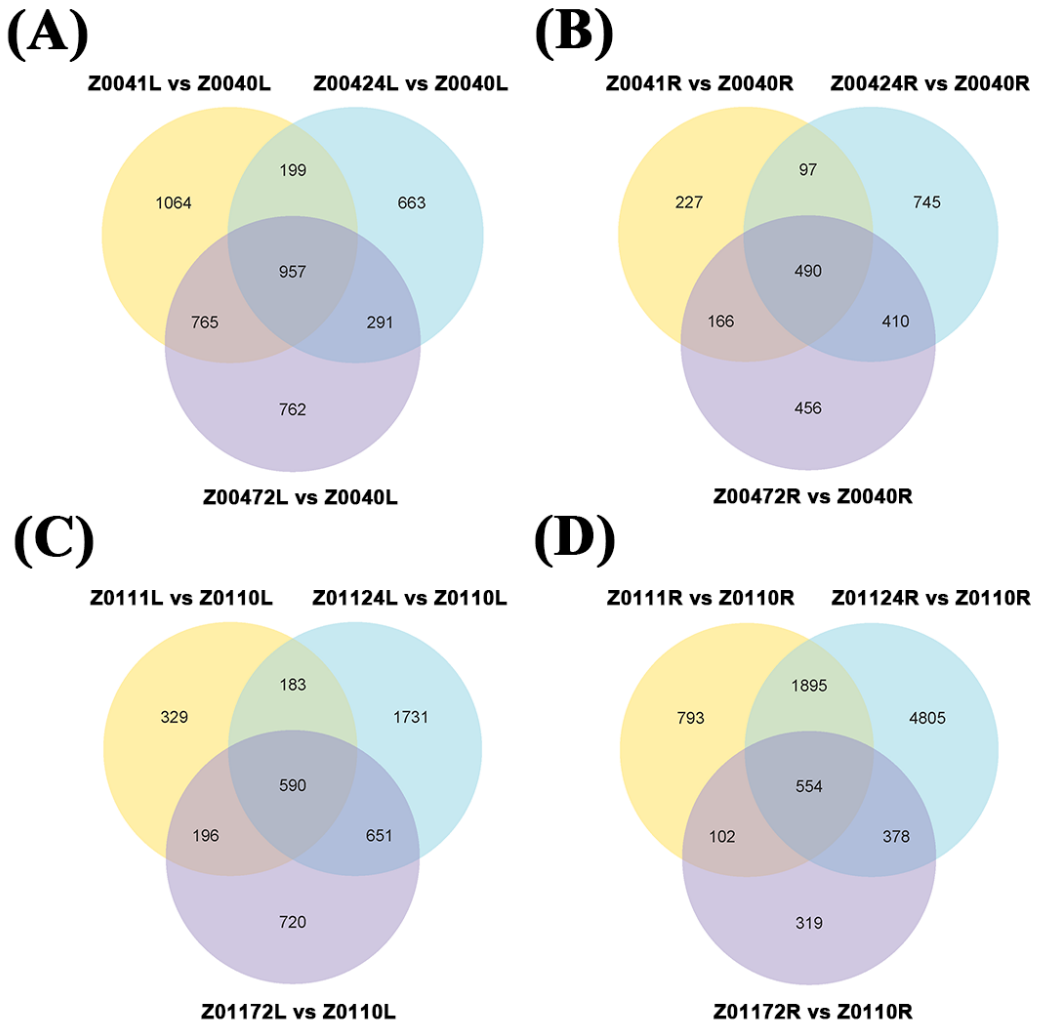

Supplementary Figure S1 Venn diagram of the number of DEGs in the leaves and roots of Z004 and Z011 after salt treatment (a) Venn diagram of the number of DEGs in Z0041L vs Z0040L, Z00424L vs Z0040L, and Z00472L vs Z0040L. (b) Venn diagram of the number of DEGs in Z0041R vs Z0040R, Z00424R vs Z0040R, and Z00472R vs Z0040R. (c) Venn diagram of the number of DEGs in Z0111L vs Z0110L, Z01124L vs Z0110L, and Z01172L vs Z0110L. (d) Venn diagram of the number of DEGs in Z0111R vs Z0110R, Z01124R vs Z0110R, and Z01172R vs Z0110R

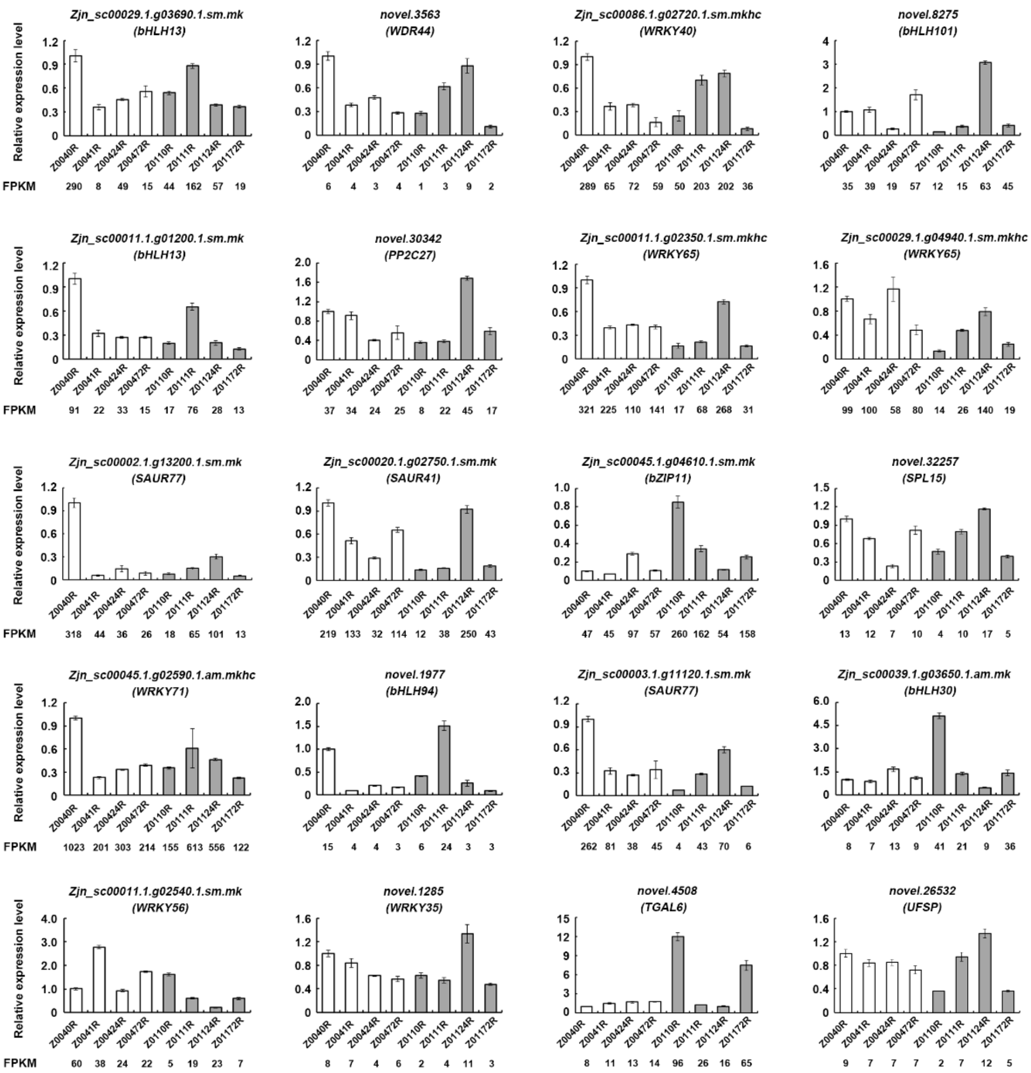

Supplementary Figure S2 qRT-PCR validation of 20 genes randomly selected from the 39 DEGs in Table 1 in Z004 and Z011 roots. The error bars indicate the SEs
