# Supplementary material for: Physiological and transcriptomic analyses reveal the mechanisms underlying the salt tolerance of Zoysia japonica Steud
Source: BMC Plant Biol. 2020 Mar 14;20:114. doi: 10.1186/s12870-020-02330-6 (PMC7071773; doi:10.1186/s12870-020-02330-6)
Supplement: Supplementary file 2 — Additional file 2: Online Resource 1 Summary of RNA-seq results and their matches to the Z. japonica genome. [file 12870_2020_2330_MOESM2_ESM.pdf]

Online Resource 1 Summary of RNA-seq results and their matches to the *Z. japonica* genome

Samples	Raw Reads	Clean Reads	Clean Bases	≥Q30	GC Content	Mapped Reads	Uniq Mapped Reads	Multiple Mapped Reads	Splice Mapped Reads
Z0040L	32438255	31524956	9.46G	94.6	55.83%	57621250 (91.39%)	56624677 (89.81%)	996573 (1.58%)	18071152 (28.66%)
Z0041L	26357459	25288464	7.59G	92.5	54.76%	45782098 (90.52%)	45017095 (89.01%)	765003 (1.51%)	16292620 (32.21%)
Z00424L	24358613	23526320	7.06G	92.71	54.56%	42663657 (90.67%)	41993576 (89.25%)	670081 (1.42%)	12985607 (27.6%)
Z00472L	31388773	30389440	9.12G	94.74	55.81%	55543678 (91.39%)	54603427 (89.84%)	940251 (1.55%)	18803898 (30.94%)
Z0040R	28748978	27331853	8.2G	94.85	56.39%	45537682 (83.31%)	44882876 (82.11%)	654806 (1.2%)	14366719 (26.28%)
Z0041R	25217276	24597846	7.38G	92.33	54.40%	30844803 (62.7%)	30401692 (61.8%)	443111 (0.9%)	10116388 (20.56%)
Z00424R	32836307	31933917	9.58G	94.73	54.30%	48700816 (76.25%)	47999420 (75.15%)	701396 (1.1%)	15287937 (23.94%)
Z00472R	36880428	35688473	10.71G	94.71	54.97%	56581667 (79.27%)	55714572 (78.06%)	867095 (1.21%)	18346344 (25.7%)
Z0110L	26586917	25922402	7.78G	92.33	54.63%	46443799 (89.58%)	45577309 (87.91%)	866490 (1.67%)	16809137 (32.42%)
Z0111L	27176365	26429692	7.93G	92.49	54.77%	47016201 (88.95%)	46171840 (87.35%)	844361 (1.6%)	16310460 (30.86%)
Z01124L	31282942	29168068	8.75G	90.33	53.57%	51780347 (88.76%)	50915346 (87.28%)	865001 (1.48%)	15892912 (27.24%)
Z01172L	29732023	27463655	8.24G	90.81	53.51%	49171344 (89.52%)	48301793 (87.94%)	869551 (1.58%)	16970162 (30.9%)
Z0110R	23872338	23011861	6.9G	93	52.11%	31773084 (69.04%)	31381515 (68.19%)	391569 (0.85%)	8092154 (17.58%)
Z0111R	31726696	30928060	9.28G	90.35	54.36%	45880277 (74.17%)	45225490 (73.11%)	654787 (1.06%)	14994212 (24.24%)
Z01124R	20666337	20135617	6.04G	91.57	56.20%	30976008 (76.92%)	30529687 (75.81%)	446321 (1.11%)	10196114 (25.32%)
Z01172R	32232323	31023161	9.31G	94.05	53.74%	42725009 (68.86%)	42118724 (67.88%)	606285 (0.98%)	13160493 (21.21%)
