# Supplementary material for: Physiological and transcriptomic analyses reveal the mechanisms underlying the salt tolerance of Zoysia japonica Steud
Source: BMC Plant Biol. 2020 Mar 14;20:114. doi: 10.1186/s12870-020-02330-6 (PMC7071773; doi:10.1186/s12870-020-02330-6)
Supplement: Supplementary file 3 — Additional file 3: Online Resource 2 The proportion of reads in the Z. japonica genomic exon, intron and intergenic regions. [file 12870_2020_2330_MOESM3_ESM.pdf]

Online Resource 2 The proportion of reads in the *Z. japonica* genomic exon region, intron region and intergenic region

Sample	Exon region	Intron region	Intergenic region
Z0040L	6586005486(76.52%)	1225282985(14.24%)	795158851(9.24%)
Z0040R	5240185581(77.08%)	911163607(13.40%)	646598688(9.51%)
Z0041L	5144175383(75.26%)	1054929622(15.43%)	636081761(9.31%)
Z0041R	3473755647(75.40%)	664988168(14.43%)	468623037(10.17%)
Z00424L	4812393510(75.52%)	912138428(14.31%)	647485365(10.16
Z00424R	5509884312(75.75%)	1035141263(14.23%)	729151280(10.02%)
Z00472L	6325682634(76.26%)	1199695922(14.46%)	769551678(9.28%)
Z00472R	6380400818(75.50%)	1237114447(14.64%)	833040473(9.86%)
Z0110L	5233660986(75.49%)	1103891679(15.92%)	595011569(8.58%)
Z0110R	3480454143(73.44%)	687012419(14.50%)	571783869(12.06%)
Z0111L	5349037833(76.22%)	1019622394(14.53%)	648792552(9.25%)
Z0111R	5222082074(76.25%)	980968822(14.32%)	645268210(9.42%)
Z01124L	5797631738(75.08%)	1136502822(14.72%)	788254073(10.21%)
Z01124R	3567519216(77.17%)	618552731(13.38%)	436917747(9.45%)
Z01172L	5440107817(74.18%)	1180720062(16.10%)	712723037(9.72%)
Z01172R	4795368758(75.22%)	928604395(14.57%)	651139166(10.21%)
