# Supplementary material for: Physiological and transcriptomic analyses reveal the mechanisms underlying the salt tolerance of Zoysia japonica Steud
Source: BMC Plant Biol. 2020 Mar 14;20:114. doi: 10.1186/s12870-020-02330-6 (PMC7071773; doi:10.1186/s12870-020-02330-6)
Supplement: Supplementary file 7 — Additional file 7: Online Resource 6 Primer sequences used for qRT-PCR. [file 12870_2020_2330_MOESM7_ESM.pdf]

Online Resource 6 Primer sequences used for qRT-PCR

Gene	Forward primer	Reverse primer
**Zjn_sc00029.1.g03690.1.sm.mk**	GTGCCTGGCTGGTTCGTC	CGGGTGTAACAGCGAGGG
**novel.3563**	GGGAGCTTTCGGAGCCTTAG	TCCGCAACCATCCGAACT
**Zjn_sc00086.1.g02720.1.sm.mkhc**	CCTCAGCCTGGTGGTGAAA	GGCCACGAGGATGGTATTGT
**novel.8275**	CTGCAAGCCAGGATTATCGA	TTCTCGAAGGTGGAGTGGGT
**Zjn_sc00011.1.g01200.1.sm.mk**	GGACTTCACCAAGCAGGAACA	CGACGCCTTGTCCATCTTG
**novel.30342**	GTCCCTTCTGGTGGCTAATGC	GTCACTGCCTATTATCAAGAACTCATC
**Zjn_sc00011.1.g02350.1.sm.mkhc**	ACTCCTTCGAGCACAACCACT	ACATCGAGCCGTACAGCATG
**Zjn_sc00029.1.g04940.1.sm.mkhc**	TTTCCGAGGGCTTACTACAGG	GGAGTGGTTGTGCTCGAAGG
**Zjn_sc00002.1.g13200.1.sm.mk**	ACGGTCGTGGTCGGGAAG	CTGGGCGTAGAACTCGATGA
**Zjn_sc00020.1.g02750.1.sm.mk**	GACTTCATGGAGGTCATCTGGG	CCGATCACGACCTGTGCC
**Zjn_sc00045.1.g04610.1.sm.mk**	CGGCTCCAGTCGCTCAAC	CGGGCAGGATTTCAAGAGG
**novel.32257**	CTGCGACTGTTTGGTAGCGT	ATGCCCGACTTGTGCTGAC
**Zjn_sc00045.1.g02590.1.am.mkhc**	GAGGGCGAGCACAACCAC	TCCCTGGTTAGCGTCATCG
**novel.1977**	CGAGACCCAGCGGATGAC	TGCGGGAAGGTGAAGAAGC
**Zjn_sc00003.1.g11120.1.sm.mk**	TCGACTCCTACCCGTTCCG	GCGTAGAACTCGATGACCTCC
**Zjn_sc00039.1.g03650.1.am.mk**	CCCCAACACCACTAAGACGG	GACGCACGCATCACAAGC
**Zjn_sc00011.1.g02540.1.sm.mk**	CCGACGATGGTGGACTGG	CTTCTTCTTCCCTGACCTCCC
**novel.1285**	GCTCGCATCAGTCGCACAT	AAATGGATAACAAATGCGAGTGA
**novel.4508**	TGTTGAACGAACGGTTTGACT	CAGGGTCTCCGTGACGTTCT
**novel.26532**	AATCCGTGGACTGTTACCGC	TCCACAGCCTCCGACACG
**ZmActin**	CACTGTGCCAATCTATGAAGGA	GAGGTTGTGAATGAGTAACCCC
