# Supplementary material for: Physiological and transcriptomic analyses reveal the mechanisms underlying the salt tolerance of Zoysia japonica Steud
Source: BMC Plant Biol. 2020 Mar 14;20:114. doi: 10.1186/s12870-020-02330-6 (PMC7071773; doi:10.1186/s12870-020-02330-6)
Supplement: Supplementary file 8 — Additional file 8: Online Resource 7 Summary of RNA-seq results and their matches to the rice (Oryza sativa L. japonica) genome. [file 12870_2020_2330_MOESM8_ESM.pdf]

Online Resource 7 Summary of RNA-seq results and their matches to the rice (*Oryza sativa L. japonica*) genome

sample	total_reads	total_map	unique_map	multi_map
Z0040L	63336298	157806(0.25%)	136347(0.22%)	21459(0.03%)
Z0041L	52075764	77325(0.15%)	65580(0.13%)	11745(0.02%)
Z00424L	47359764	117744(0.25%)	97778(0.21%)	19966(0.04%)
Z00472L	61159122	171959(0.28%)	143925(0.24%)	28034(0.05%)
Z0040R	55216010	154858(0.28%)	142119(0.26%)	12739(0.02%)
Z0041R	49248370	90947(0.18%)	82930(0.17%)	8017(0.02%)
Z00424R	64937550	78662(0.12%)	71043(0.11%)	7619(0.01%)
Z00472R	71905504	176499(0.25%)	160202(0.22%)	16297(0.02%)
Z0110L	51926614	129285(0.25%)	105645(0.2%)	23640(0.05%)
Z0111L	52839672	203465(0.39%)	133970(0.25%)	69495(0.13%)
Z01124L	58726026	138042(0.24%)	116377(0.2%)	21665(0.04%)
Z01172L	55978756	138753(0.25%)	117203(0.21%)	21550(0.04%)
Z0110R	46133940	88561(0.19%)	74135(0.16%)	14426(0.03%)
Z0111R	62038566	149695(0.24%)	136037(0.22%)	13658(0.02%)
Z01124R	40325160	111764(0.28%)	102971(0.26%)	8793(0.02%)
Z01172R	62536392	125893(0.2%)	111967(0.18%)	13926(0.02%)
