# Supplementary material for: Physiological and transcriptomic analyses reveal the mechanisms underlying the salt tolerance of Zoysia japonica Steud
Source: BMC Plant Biol. 2020 Mar 14;20:114. doi: 10.1186/s12870-020-02330-6 (PMC7071773; doi:10.1186/s12870-020-02330-6)
Supplement: Supplementary file 9 — Additional file 9: Online Resource 8 Summary of RNA-seq results and their matches to the Sorghum bicolor genome. [file 12870_2020_2330_MOESM9_ESM.pdf]

Online Resource 8 Summary of RNA-seq results and their matches to the *Sorghum bicolor* genome

sample	total_reads	total_map	unique_map	multi_map
Z0040L	63336298	416658(0.66%)	402227(0.64%)	14431(0.02%)
Z0041L	52075764	160397(0.31%)	147668(0.28%)	12729(0.02%)
Z00424L	47359764	293739(0.62%)	274982(0.58%)	18757(0.04%)
Z00472L	61159122	429494(0.7%)	397574(0.65%)	31920(0.05%)
Z0040R	55216010	351190(0.64%)	342866(0.62%)	8324(0.02%)
Z0041R	49248370	201600(0.41%)	196073(0.4%)	5527(0.01%)
Z00424R	64937550	170588(0.26%)	166274(0.26%)	4314(0.01%)
Z00472R	71905504	374855(0.52%)	364169(0.51%)	10686(0.01%)
Z0110L	51926614	313930(0.6%)	296944(0.57%)	16986(0.03%)
Z0111L	52839672	451372(0.85%)	380557(0.72%)	70815(0.13%)
Z01124L	58726026	337138(0.57%)	316551(0.54%)	20587(0.04%)
Z01172L	55978756	352907(0.63%)	333271(0.6%)	19636(0.04%)
Z0110R	46133940	178257(0.39%)	169858(0.37%)	8399(0.02%)
Z0111R	62038566	345637(0.56%)	336543(0.54%)	9094(0.01%)
Z01124R	40325160	232601(0.58%)	227155(0.56%)	5446(0.01%)
Z01172R	62536392	306199(0.49%)	297283(0.48%)	8916(0.01%)
